# 

**Published:** 1895-12

**Authors:** 


					CONTENTS.
-
School Surgery.
By Arthur W.
Prichard, M.R.C.S. Eng.
241
Two Cases of Fibroid Degeneration
of the Myocardium.
ByTHEODORE
Fisher, M.D. Lond.
258
Notes on Egypt as a Health-Resort.
By P. Watson Williams, M.D.
Lond.
263
Progress of the Medical Sciences:
Medicine.
By J. Michell Clarke, j
M.D.
273
Surgery.
By W. H. Harsant
... 280
Laryngology.
By BarclayJ. Baron,
M.B.
286
Otology.
By W. H. Harsant,
Reviews of Books
292
Notes on Preparations for the Sick
309
Bristol Medico-Chlrurglcal Society
312
The Library of the Bristol Medico-
Chirurgical Society 316
Local Medical Notes
320
Scraps

				

